# 1-[1,4-Bis(but-3-en-1-yl­oxy)]-2,3,4,5-(1,4-dimeth­oxy)pillar[5]arene–1,4-di­bromo­butane 1:1 inclusion complex

**DOI:** 10.1107/S2414314623005886

**Published:** 2023-07-14

**Authors:** Mickey Vinodh, Talal F. Al-Azemi

**Affiliations:** a Department of Chemistry, Kuwait University, PO Box 5969, Safat 13060, Kuwait; Zhejiang University (Yuquan Campus), China

**Keywords:** crystal structure, A1/A2-dibuten­oxy pillar[5]arene, di­bromo­butane, host–guest system

## Abstract

In the title compound, C_51_H_58_O_10_·C_4_H_8_Br_2_, both the host and guest are completed by crystallographic twofold symmetry (one carbon atom of the host lies on the rotation axis). The penta­gonal-shaped macrocycle has a pair of butene­oxy substituents on one of its faces and one mol­ecule of 1,4-di­bromo­butane is encapsulated within the cavity of the pillararene, forming a 1:1 inclusion complex. The terminal alkene parts, which project outwards from the pillararene ring, exhibit positional disorder over two sets of sites in a 0.52 (2): 0.48 (2) ratio. The host and guest inter­act *via* C—H⋯O, C—H⋯Br and C—H⋯π inter­actions and adjacent host mol­ecules inter­act *via* C—H⋯O and C—H⋯π bonds.

## Abstract 2

In the title compound, both the host and guest are completed by crystallographic twofold symmetry (one carbon atom of the host lies on the rotation axis). The penta­gonal-shaped macrocycle has a pair of butene­oxy substituents on one of its faces and one mol­ecule of 1,4-di­bromo­butane is encapsulated within the cavity of the pillararene, forming a 1:1 inclusion complex.

## Graphical abstract

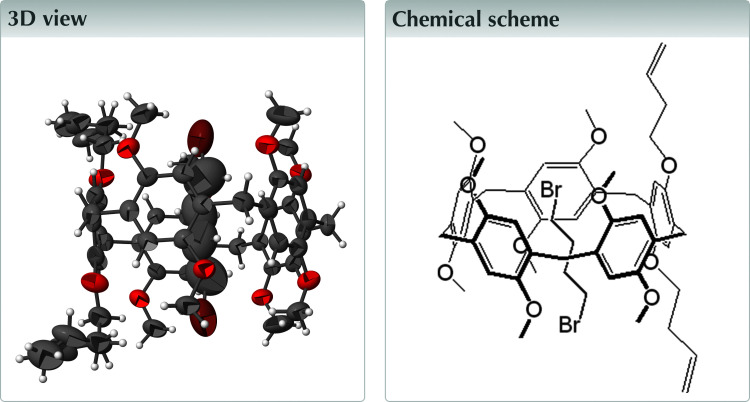

## Structure description

Pillar[*n*]arenes are characterized by guest encapsulation and mol­ecular recognition properties, which are due to their pillar-shaped structures, nano-sized cavities and availability of multiple rim sites for substitutions, and which makes them useful functional materials for several applications in materials chemistry, nanotechnology and biomimmetic systems (Ogoshi *et al.*, 2016 ▸; Li *et al.*, 2020 ▸). Appropriate derivatization of pillararene macrocycles can be achieved by selective functionalization of pillararene rims (Zhang *et al.*, 2021 ▸; Al-Azemi & Vinodh, 2022 ▸; Vinodh *et al.*, 2023 ▸). Selective derivatization of pillarene rims enables self-assembly of these macromolecules to form supra­molecular polymers or make them capable of inter­acting with flexible binding sites, for example proteins (Liu *et al.*, 2023 ▸). The suitably functionalized pillarenenes could conjugate with other functional units such as drug moieties or photosensitizing agents and might generate potentially useful functional materials for a variety of applications such as drug delivery, light harvesting systems, sensors, detection and separation (Feng *et al.*, 2017 ▸; Kakuta *et al.*, 2018 ▸; Hua *et al.*, 2020 ▸; Khalil-Cruz *et al.*, 2021 ▸).

In the present work, an inclusion system comprising buten­oxy-substituted pillararene and di­bromo­butane is reported. The parent pillararene-1-[1–4-di(but-3-en-1-yl­oxy)]-2,3,4,5-(1,4-dimeth­oxy)pillar[5]arene [**Pil(Buten­oxy)2**] exhibits butene­oxy substitution at both ends of its macrocyclic rims. Single crystals of this pillararene were grown from a solution containing di­bromo­butane and its structural as well as supra­molecular features are discussed.

**Figure 1:**
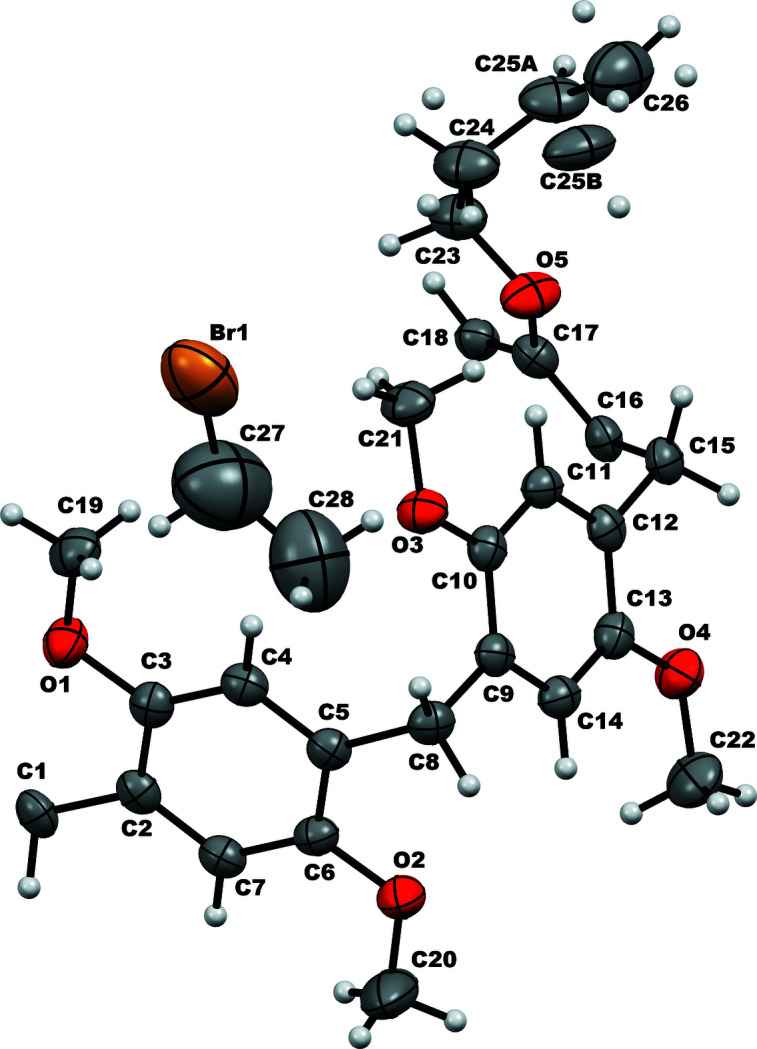
Displacement ellipsoid representation (30% probability) of the asymmetric unit of **Pil(Buten­oxy)2·ButBr2**.

**Figure 2:**
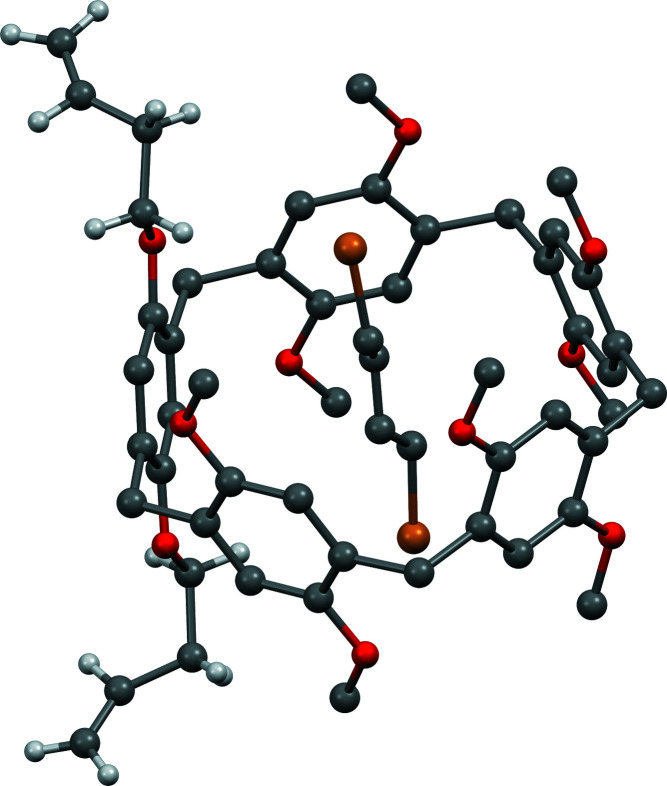
Crystal structure of **Pil(Buten­oxy)2·ButBr2** after symmetry expansion. Hydrogen atoms, except those of the butene substituent of the pillararene, are omitted for clarity.

The inclusion complex crystallizes in the monoclinic crystal system, space group *C*2/*c*. The asymmetric unit contains half of the pillararene mol­ecule (Fig. 1 ▸) and half the guest molecule. The complete structure (Fig. 2 ▸) is obtained by symmetry expansion *via* crystallographic twofold axes. In the crystal, one mol­ecule of di­bromo­butane is encapsulated within the cavity of the pillararene. The terminal alkene parts, which project outwards from the pillararene ring, exhibit positional disorder. As a result, the exact orientation of the vinyl groups with respect to the pillararene macrocycle could not be obtained from the crystal data. In Fig. 2 ▸ the orientation of the major occupancy butene component is shown.

**Figure 3:**
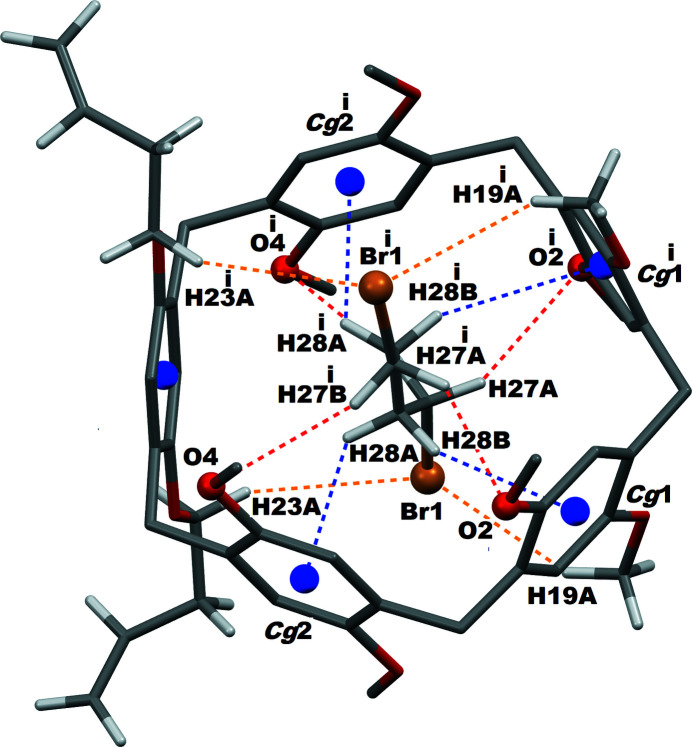
Non-bonding inter­actions between the pillararene macrocycle host and di­bromo­butane guest in **Pil(Buten­oxy)2·ButBr2** crystals. C—H⋯O inter­actions are represented by red, C—H⋯Br by orange and C—H⋯π by blue dashed lines. *Cg*1 and *Cg*2 are the centroids of the pillararene rings C2–C7 and C9–C13, respectively. Symmetry code: (i) −*x*, *y*, 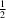 − *z*.

The crystal structure of **Pil(Buten­oxy)2·ButBr2** shows the that 1,4-di­bromo­butane guest species is threaded inside the pillararene cavity, forming a 1:1 inclusion complex. All of the H atoms of the guest mol­ecule are capable of engaging in non-bonding inter­actions with pillararene ring, either *via* C—H⋯O or C—H⋯π inter­actions. In addition, the pillararene macrocycle is able to connect with the bromine atoms of the di­bromo­butane by C—H⋯Br inter­actions. The nature of these various non-bonding inter­actions are depicted in Fig. 3 ▸ and their qu­anti­tative details are provided in Table 1 ▸.

**Figure 4:**
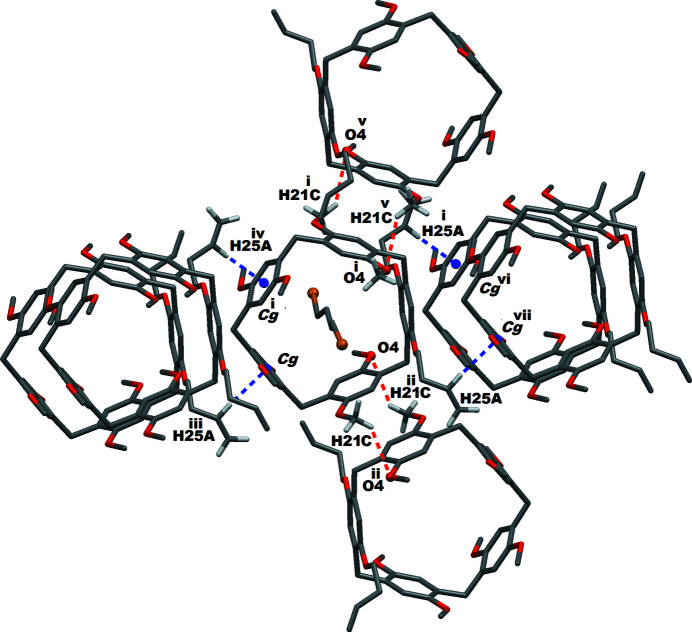
Inter­molecular non-bonding inter­actions between the pillararene macrocycle and its neighboring counterparts. C—H⋯O inter­actions are represented by red and C—H⋯π by blue dashed lines. *Cg*1 is the centroid of the pillararene phenyl ring C2–C7. Symmetry codes: (i) −*x*, *y*, 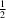 − *z*; (ii) 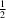 − *x*, 1.5 − *y*, 1 − *z*; (iii) 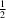 + *x*, − 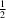 + *y*, *z*; (iv) − 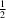 − *x*, − 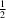 + *y*, 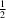 − *z*; (v) − 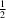 + *x*, 1.5 − *y*, − 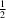 + *z*, (vi) − 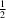 − *x*, 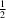 + *y*, 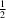 − *z*; (vii) − 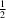 + *x*, 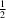 + *y*, *z*.

**Figure 5:**
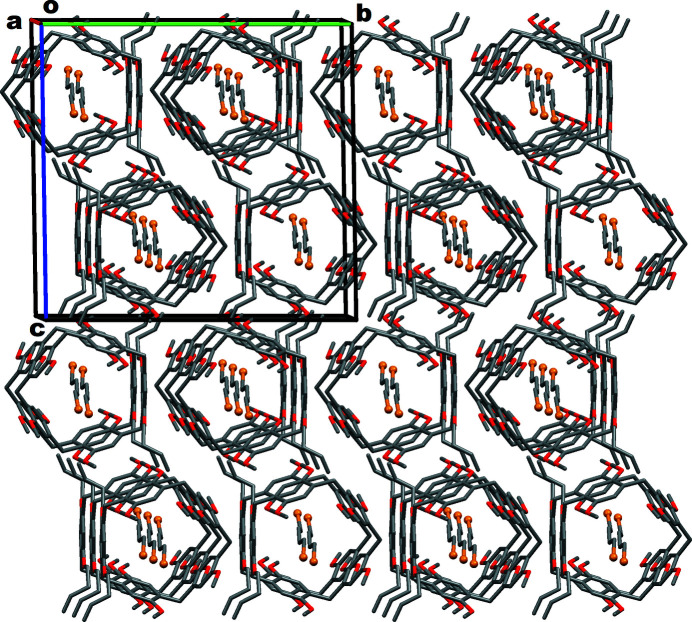
Packing pattern of **Pil(Buten­oxy)2·ButBr2** crystals.

The **Pil(Buten­oxy)2·ButBr2** species exhibit inter­molecular non-bonding C—H⋯O or C—H⋯π inter­actions in their crystal network. The multiple non-bonding (non-covalent/non-coordinate) inter­actions (less than the van der Waals range) between neighboring **Pil(Buten­oxy)2.ButBr2** systems are shown in Fig. 4 ▸. It can be seen that each pillararene unit inter­acts with six immediate neighboring pillararenes in its crystal network. The packing pattern of the **Pil(Buten­oxy)2·ButBr2** complex is depicted in Fig. 5 ▸, which shows that the crystal network forms one-dimensional channels along the *a*-axis direction.

## Synthesis and crystallization

Synthesis of vinyl-substituted pillararene **Pil(Buten­oxy)2**: Paraformaldehyde (930 mg, 30 mmol) was added to a solution of 1,4-di­meth­oxy­benzene (1.10 g, 8 mmol) and 1,4-bis­(but-3-en-1-yl­oxy)benzene (436 mg, 2 mmol) in 1,2-di­chloro­ethane (60 ml) under a nitro­gen atmosphere. Boron trifluoride diethyl etherate (1.25 ml, 10 mmol) was then added to the solution and the mixture was stirred at 0°C for 1 h. MeOH (200 ml) was poured into the mixture to quench the reaction and the reaction mixture was filtered. The residue was dissolved in chloro­form (50 mL) and filtered. The filtrate was concentrated to a small volume and adsorbed on silica and column chromatography was performed using a di­chloro­methane:hexane mixture (60:40 *v*/*v*). The second last fraction was the intended pillarene. Yield: 228 mg (16%). ^1^H NMR (400 MHz, CDCl_3_,) δ: 2.50 (*m*, 4H), 3.68 (*m*, 24H) 3.80 (*m*, 10H), 3.91 (*t*, *J* = 6.8 & *J* = 6.4 Hz, 4H), 5.08 (*m*, 4H), 5.92 (*m*, 2H), 6.79 (*m*, 10H). ^13^C NMR (150 MHz, CDCl3), δ: 29.8, 29.8, 29.9, 34.4, 56.0, 56.0, 56.0, 56.1, 68.0, 114.3, 114.3, 114.4, 114.4, 115.4, 116.9, 128.3, 128.4, 128.5, 128.6, 128.6, 135.2, 150.1, 151.0, 151.0, 151.0.

Crystal growth of **Pil(Buten­oxy)2·ButBr2** inclusion complex: **Pil(Buten­oxy)2** (20 mg) was dissolved in a solution of di­chloro­methane and 1,4 di­bromo butane (90: 10; *v*/*v*, 1 mL). Single crystals of pillararene encapsulated with the di­bromo­butane guest were grown by slow solvent evaporation after storing the solution in an NMR tube that was kept cold. Crystals suitable for X-ray diffraction were grown in 5 days.

## Refinement

Crystal data, data collection and structure refinement details are summarized in Table 2 ▸. The vinyl site exhibits positional disorder and thus was refined over two sets of sites with a 0.52 (2):0.48 (2) occupancy ratio.

## Supplementary Material

Crystal structure: contains datablock(s) I. DOI: 10.1107/S2414314623005886/xu4051sup1.cif

Supporting information file. DOI: 10.1107/S2414314623005886/xu4051Isup3.cdx

Structure factors: contains datablock(s) I. DOI: 10.1107/S2414314623005886/xu4051Isup4.hkl

CCDC reference: 2254104

Additional supporting information:  crystallographic information; 3D view; checkCIF report

## Figures and Tables

**Table 1:** Non-bonding inter­actions (Å, °) between the pillararene host and di­bromo­butane guest in **Pil(Buten­oxy)2·ButBr2** *Cg*1 and *Cg*2 are the centroids of the C2–C7 and C9–C13 rings, respectively.

*D*—H⋯*A*	*D*—H	H⋯*A*	*D*⋯*A*	*D*—H⋯*A*
C27—H27*A*⋯O2^i^	0.97	3.06	3.82 (1)	136
C27—H27*B*⋯O4^i^	0.97	3.06	3.99 (1)	160
C28—H28*B*⋯*Cg*1	0.97	3.10	4.015	158
C28—H28*A*⋯*Cg*2	0.97	3.28	3.859	120
C19—H19*A*⋯Br1	0.96	3.14	3.968 (5)	145
C23—H23*A*⋯Br1	0.97	3.15	4.039 (5)	154

Symmetry code: (i) −*x*, *y*, 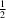 − *z*.

**Table 2:** Experimental details

Crystal data	
Chemical formula	C_51_H_58_O_10_·C_4_H_8_Br_2_
*M*_r_	1046.89
Crystal system, space group	Monoclinic, *C*2/*c*
Temperature (K)	293
*a*, *b*, *c* (Å)	11.3071 (12), 22.044 (3), 21.557 (3)
β (°)	104.775 (7)
*V* (Å^3^)	5195.4 (11)
*Z*	4
Radiation type	Mo *K*α
μ (mm^−1^)	1.62
Crystal size (mm)	0.21 × 0.18 × 0.17

Data collection	
Diffractometer	Rigaku R-AXIS RAPID
Absorption correction	Multi-scan (*ABSCOR*; Higashi, 1995 ▸)
*T*_min_, *T*_max_	0.449, 0.723
No. of measured, independent and observed [*I* > 2σ(*I*)] reflections	16532, 4576, 2385
*R*_int_	0.055
(sin θ/λ)_max_ (Å^−1^)	0.595

Refinement	
*R*[*F* ^2^ > 2σ(*F* ^2^)], *wR*(*F* ^2^), *S*	0.073, 0.251, 1.05
No. of reflections	4576
No. of parameters	317
No. of restraints	53
H-atom treatment	H-atom parameters constrained
Δρ_max_, Δρ_min_ (e Å^−3^)	0.68, −0.60

Computer programs: *CrystalClear* (Rigaku, 2016 ▸), *CrystalStructure* (Rigaku, 2017 ▸), *SHELXL2017/1* (Sheldrick, 2015 ▸) and *Mercury* (Macrae *et al.*, 2020 ▸).

## full crystallographic data

### Crystal data

**Table:**

C_51_H_58_O_10_·C_4_H_8_Br_2_	*F*(000) = 2184
*M**_r_* = 1046.89	*D*_x_ = 1.338 Mg m^−^^3^
Monoclinic, *C*2/*c*	Mo *K*α radiation, λ = 0.71075 Å
*a* = 11.3071 (12) Å	Cell parameters from 8137 reflections
*b* = 22.044 (3) Å	θ = 3.2–25.0°
*c* = 21.557 (3) Å	µ = 1.62 mm^−^^1^
β = 104.775 (7)°	*T* = 293 K
*V* = 5195.4 (11) Å^3^	Block, colorless
*Z* = 4	0.21 × 0.18 × 0.17 mm

### Data collection

**Table:**

Rigaku R-AXIS RAPID diffractometer	2385 reflections with *I* > 2σ(*I*)
Detector resolution: 10.000 pixels mm^-1^	*R*_int_ = 0.055
ω scans	θ_max_ = 25.0°, θ_min_ = 3.2°
Absorption correction: multi-scan (ABSCOR; Higashi, 1995)	*h* = −13→13
*T*_min_ = 0.449, *T*_max_ = 0.723	*k* = −26→25
16532 measured reflections	*l* = −25→25
4576 independent reflections	

### Refinement

**Table:**

Refinement on *F*^2^	53 restraints
Least-squares matrix: full	Hydrogen site location: inferred from neighbouring sites
*R*[*F*^2^ > 2σ(*F*^2^)] = 0.073	H-atom parameters constrained
*wR*(*F*^2^) = 0.251	*w* = 1/[σ^2^(*F*_o_^2^) + (0.1359*P*)^2^ + 2.4202*P*] where *P* = (*F*_o_^2^ + 2*F*_c_^2^)/3
*S* = 1.05	(Δ/σ)_max_ = 0.001
4576 reflections	Δρ_max_ = 0.68 e Å^−^^3^
317 parameters	Δρ_min_ = −0.60 e Å^−^^3^

### Special details

**Table:**

Geometry. All esds (except the esd in the dihedral angle between two l.s. planes) are estimated using the full covariance matrix. The cell esds are taken into account individually in the estimation of esds in distances, angles and torsion angles; correlations between esds in cell parameters are only used when they are defined by crystal symmetry. An approximate (isotropic) treatment of cell esds is used for estimating esds involving l.s. planes.

### Fractional atomic coordinates and isotropic or equivalent isotropic displacement parameters (Å^2^)

**Table:**

	*x*	*y*	*z*	*U*_iso_*/*U*_eq_	Occ. (<1)
Br1	−0.25218 (12)	0.64113 (4)	0.32051 (5)	0.1711 (6)	
O1	−0.0929 (3)	0.45851 (15)	0.34993 (15)	0.0718 (9)	
O2	0.3781 (3)	0.52946 (16)	0.35646 (16)	0.0769 (10)	
O3	0.0972 (3)	0.63736 (13)	0.48639 (15)	0.0648 (8)	
O4	0.3667 (3)	0.76181 (15)	0.34725 (16)	0.0750 (9)	
O5	−0.0766 (3)	0.82587 (17)	0.36144 (15)	0.0791 (10)	
C1	0.000000	0.4179 (3)	0.250000	0.0591 (16)	
H1A	0.054453	0.392019	0.233599	0.071*	0.5
H1B	−0.054451	0.392016	0.266399	0.071*	0.5
C2	0.0751 (4)	0.45651 (17)	0.30448 (19)	0.0530 (11)	
C3	0.0259 (4)	0.47716 (19)	0.3536 (2)	0.0546 (11)	
C4	0.0934 (4)	0.51343 (19)	0.40126 (19)	0.0547 (11)	
H4	0.058900	0.526575	0.433728	0.066*	
C5	0.2123 (4)	0.53122 (19)	0.40247 (19)	0.0524 (10)	
C6	0.2612 (4)	0.51070 (19)	0.3538 (2)	0.0543 (11)	
C7	0.1937 (4)	0.47396 (19)	0.3057 (2)	0.0558 (11)	
H7	0.228494	0.460602	0.273450	0.067*	
C8	0.2824 (4)	0.57300 (19)	0.45434 (19)	0.0558 (11)	
H8A	0.369258	0.565007	0.461801	0.067*	
H8B	0.259341	0.564872	0.493902	0.067*	
C28	−0.0278 (9)	0.6452 (6)	0.2743 (5)	0.223 (5)	
H28A	−0.010730	0.684107	0.295800	0.268*	
H28B	0.012448	0.614636	0.304815	0.268*	
C27	−0.1638 (9)	0.6345 (7)	0.2617 (6)	0.267 (6)	
H27A	−0.177889	0.593711	0.244509	0.320*	
H27B	−0.201915	0.661661	0.226904	0.320*	
C9	0.2575 (4)	0.63911 (18)	0.43615 (18)	0.0501 (10)	
C10	0.1619 (4)	0.66987 (19)	0.45168 (18)	0.0509 (10)	
C11	0.1366 (4)	0.72954 (19)	0.43243 (18)	0.0513 (10)	
H11	0.071977	0.749348	0.443211	0.062*	
C12	0.2046 (4)	0.76038 (19)	0.39767 (18)	0.0523 (11)	
C13	0.3010 (4)	0.7291 (2)	0.38255 (19)	0.0551 (11)	
C14	0.3266 (4)	0.6697 (2)	0.40133 (18)	0.0546 (11)	
H14	0.391175	0.649902	0.390525	0.065*	
C15	0.1745 (4)	0.82511 (18)	0.37545 (19)	0.0565 (11)	
H15A	0.141270	0.846205	0.406701	0.068*	
H15B	0.249198	0.845675	0.373242	0.068*	
C16	0.0844 (4)	0.82833 (17)	0.3111 (2)	0.0530 (10)	
C17	−0.0420 (4)	0.82739 (19)	0.3050 (2)	0.0565 (11)	
C18	−0.1238 (4)	0.82843 (18)	0.2447 (2)	0.0567 (11)	
H18	−0.207327	0.829211	0.241798	0.068*	
C19	−0.1486 (4)	0.4797 (2)	0.3966 (3)	0.0786 (15)	
H19A	−0.147798	0.523234	0.396874	0.094*	
H19B	−0.104722	0.464842	0.437932	0.094*	
H19C	−0.231599	0.465546	0.387089	0.094*	
C20	0.4421 (6)	0.5009 (4)	0.3194 (4)	0.148 (3)	
H20A	0.512091	0.524929	0.317782	0.178*	
H20B	0.390682	0.495883	0.276748	0.178*	
H20C	0.468504	0.461914	0.337399	0.178*	
C21	−0.0040 (5)	0.6651 (2)	0.5023 (3)	0.0757 (14)	
H21A	−0.037779	0.637836	0.527915	0.091*	
H21B	−0.065069	0.674761	0.463673	0.091*	
H21C	0.022265	0.701684	0.526037	0.091*	
C22	0.4686 (5)	0.7339 (3)	0.3341 (3)	0.0963 (18)	
H22A	0.523491	0.720849	0.373577	0.116*	
H22B	0.509721	0.762276	0.313039	0.116*	
H22C	0.442666	0.699473	0.306815	0.116*	
C23	−0.1994 (5)	0.8196 (2)	0.3605 (3)	0.0793 (15)	
H23A	−0.231851	0.782825	0.337763	0.095*	
H23B	−0.245418	0.853786	0.338201	0.095*	
C24	−0.2120 (6)	0.8169 (3)	0.4281 (3)	0.0955 (17)	
H24A	−0.292125	0.801033	0.427760	0.115*	0.52 (2)
H24B	−0.151273	0.789161	0.452704	0.115*	0.52 (2)
H24C	−0.195555	0.775644	0.443468	0.115*	0.48 (2)
H24D	−0.296359	0.825823	0.427312	0.115*	0.48 (2)
C25A	−0.1971 (19)	0.8745 (7)	0.4588 (7)	0.095 (4)	0.52 (2)
H25A	−0.232509	0.904572	0.429708	0.114*	0.52 (2)
C25B	−0.1269 (19)	0.8609 (9)	0.4785 (8)	0.107 (4)	0.48 (2)
H25B	−0.043555	0.857046	0.482016	0.128*	0.48 (2)
C26	−0.1525 (7)	0.8967 (5)	0.5111 (4)	0.143 (3)	
H26A	−0.113530	0.872088	0.545275	0.171*	0.52 (2)
H26B	−0.157274	0.938346	0.517050	0.171*	0.52 (2)
H26C	−0.234134	0.903196	0.510285	0.171*	0.48 (2)
H26D	−0.091610	0.919371	0.538564	0.171*	0.48 (2)

### Atomic displacement parameters (Å^2^)

**Table:**

	*U*^11^	*U*^22^	*U*^33^	*U*^12^	*U*^13^	*U*^23^
Br1	0.2090 (13)	0.1184 (8)	0.1686 (10)	0.0308 (6)	0.0165 (8)	0.0075 (6)
O1	0.065 (2)	0.083 (2)	0.070 (2)	−0.0149 (17)	0.0230 (17)	−0.0046 (17)
O2	0.064 (2)	0.084 (2)	0.091 (2)	−0.0068 (17)	0.0342 (18)	−0.0205 (19)
O3	0.0680 (19)	0.068 (2)	0.065 (2)	0.0038 (15)	0.0303 (16)	0.0088 (15)
O4	0.075 (2)	0.076 (2)	0.081 (2)	−0.0089 (18)	0.0337 (18)	0.0063 (18)
O5	0.072 (2)	0.117 (3)	0.0510 (19)	0.012 (2)	0.0212 (16)	−0.0046 (18)
C1	0.076 (4)	0.039 (3)	0.060 (4)	0.000	0.012 (3)	0.000
C2	0.065 (3)	0.044 (2)	0.046 (2)	0.005 (2)	0.008 (2)	0.0071 (19)
C3	0.059 (3)	0.052 (2)	0.051 (3)	0.001 (2)	0.011 (2)	0.010 (2)
C4	0.063 (3)	0.056 (2)	0.045 (2)	0.006 (2)	0.015 (2)	0.003 (2)
C5	0.056 (3)	0.052 (2)	0.046 (2)	0.005 (2)	0.0087 (19)	0.0022 (19)
C6	0.052 (3)	0.056 (2)	0.058 (3)	0.005 (2)	0.020 (2)	0.005 (2)
C7	0.066 (3)	0.051 (2)	0.052 (2)	0.007 (2)	0.018 (2)	0.000 (2)
C8	0.054 (2)	0.065 (3)	0.046 (2)	0.007 (2)	0.0070 (19)	0.004 (2)
C28	0.273 (10)	0.216 (10)	0.163 (11)	−0.040 (12)	0.026 (9)	0.008 (7)
C27	0.279 (12)	0.300 (14)	0.218 (11)	0.030 (13)	0.058 (10)	−0.038 (10)
C9	0.049 (2)	0.056 (2)	0.041 (2)	−0.001 (2)	0.0029 (18)	−0.0063 (19)
C10	0.053 (2)	0.059 (3)	0.038 (2)	−0.007 (2)	0.0077 (18)	−0.0027 (19)
C11	0.047 (2)	0.060 (3)	0.044 (2)	0.0011 (19)	0.0057 (18)	−0.005 (2)
C12	0.058 (3)	0.056 (2)	0.037 (2)	−0.005 (2)	0.0001 (19)	−0.0074 (19)
C13	0.056 (3)	0.066 (3)	0.043 (2)	−0.012 (2)	0.0128 (19)	−0.003 (2)
C14	0.050 (2)	0.066 (3)	0.045 (2)	0.001 (2)	0.0080 (19)	−0.007 (2)
C15	0.060 (2)	0.054 (2)	0.051 (2)	−0.008 (2)	0.006 (2)	−0.009 (2)
C16	0.062 (3)	0.040 (2)	0.054 (3)	−0.005 (2)	0.009 (2)	−0.0014 (19)
C17	0.069 (3)	0.050 (2)	0.050 (3)	0.005 (2)	0.015 (2)	−0.001 (2)
C18	0.057 (3)	0.054 (2)	0.058 (3)	0.007 (2)	0.012 (2)	−0.002 (2)
C19	0.068 (3)	0.084 (3)	0.092 (4)	−0.005 (3)	0.036 (3)	0.010 (3)
C20	0.084 (4)	0.173 (7)	0.209 (8)	−0.027 (5)	0.077 (5)	−0.089 (7)
C21	0.072 (3)	0.085 (3)	0.081 (3)	0.004 (3)	0.041 (3)	0.002 (3)
C22	0.091 (4)	0.109 (5)	0.105 (4)	−0.015 (3)	0.054 (4)	0.007 (4)
C23	0.081 (4)	0.083 (4)	0.082 (4)	0.013 (3)	0.033 (3)	0.003 (3)
C24	0.105 (4)	0.103 (4)	0.094 (4)	0.017 (3)	0.054 (3)	0.010 (3)
C25A	0.101 (9)	0.124 (7)	0.069 (6)	0.030 (7)	0.039 (6)	−0.003 (6)
C25B	0.076 (8)	0.166 (10)	0.083 (8)	0.037 (7)	0.028 (6)	−0.011 (6)
C26	0.110 (5)	0.185 (8)	0.125 (6)	0.004 (5)	0.016 (5)	−0.032 (5)

### Geometric parameters (Å, º)

**Table:**

Br1—C27	1.810 (9)	C12—C15	1.516 (6)
O1—C3	1.388 (5)	C13—C14	1.380 (6)
O1—C19	1.397 (5)	C14—H14	0.9300
O2—C20	1.361 (7)	C15—C16	1.498 (6)
O2—C6	1.371 (5)	C15—H15A	0.9700
O3—C10	1.374 (5)	C15—H15B	0.9700
O3—C21	1.415 (5)	C16—C18^i^	1.387 (6)
O4—C13	1.392 (5)	C16—C17	1.402 (6)
O4—C22	1.397 (6)	C17—C18	1.389 (6)
O5—C17	1.371 (5)	C18—H18	0.9300
O5—C23	1.389 (6)	C19—H19A	0.9600
C1—C2^i^	1.522 (5)	C19—H19B	0.9600
C1—C2	1.522 (5)	C19—H19C	0.9600
C1—H1A	0.9700	C20—H20A	0.9600
C1—H1B	0.9700	C20—H20B	0.9600
C2—C7	1.389 (6)	C20—H20C	0.9600
C2—C3	1.393 (6)	C21—H21A	0.9600
C3—C4	1.369 (6)	C21—H21B	0.9600
C4—C5	1.394 (6)	C21—H21C	0.9600
C4—H4	0.9300	C22—H22A	0.9600
C5—C6	1.382 (6)	C22—H22B	0.9600
C5—C8	1.508 (6)	C22—H22C	0.9600
C6—C7	1.382 (6)	C23—C24	1.503 (7)
C7—H7	0.9300	C23—H23A	0.9700
C8—C9	1.517 (6)	C23—H23B	0.9700
C8—H8A	0.9700	C24—C25A	1.421 (15)
C8—H8B	0.9700	C24—C25B	1.585 (19)
C28—C28^i^	1.354 (17)	C24—H24A	0.9700
C28—C27	1.510 (5)	C24—H24B	0.9700
C28—H28A	0.9700	C24—H24C	0.9700
C28—H28B	0.9700	C24—H24D	0.9700
C27—H27A	0.9700	C25A—C26	1.216 (16)
C27—H27B	0.9700	C25A—H25A	0.9300
C9—C10	1.387 (6)	C25B—C26	1.142 (16)
C9—C14	1.389 (6)	C25B—H25B	0.9300
C10—C11	1.387 (6)	C26—H26A	0.9300
C11—C12	1.382 (6)	C26—H26B	0.9300
C11—H11	0.9300	C26—H26C	0.9300
C12—C13	1.396 (6)	C26—H26D	0.9300
			
C3—O1—C19	117.8 (4)	C12—C15—H15A	109.1
C20—O2—C6	119.1 (4)	C16—C15—H15B	109.1
C10—O3—C21	118.8 (4)	C12—C15—H15B	109.1
C13—O4—C22	117.7 (4)	H15A—C15—H15B	107.8
C17—O5—C23	119.9 (4)	C18^i^—C16—C17	117.6 (4)
C2^i^—C1—C2	112.0 (4)	C18^i^—C16—C15	120.7 (4)
C2^i^—C1—H1A	109.2	C17—C16—C15	121.6 (4)
C2—C1—H1A	109.2	O5—C17—C18	123.9 (4)
C2^i^—C1—H1B	109.2	O5—C17—C16	115.5 (4)
C2—C1—H1B	109.2	C18—C17—C16	120.6 (4)
H1A—C1—H1B	107.9	C16^i^—C18—C17	121.8 (4)
C7—C2—C3	117.8 (4)	C16^i^—C18—H18	119.1
C7—C2—C1	121.1 (4)	C17—C18—H18	119.1
C3—C2—C1	121.1 (4)	O1—C19—H19A	109.5
C4—C3—O1	124.2 (4)	O1—C19—H19B	109.5
C4—C3—C2	120.5 (4)	H19A—C19—H19B	109.5
O1—C3—C2	115.3 (4)	O1—C19—H19C	109.5
C3—C4—C5	121.9 (4)	H19A—C19—H19C	109.5
C3—C4—H4	119.1	H19B—C19—H19C	109.5
C5—C4—H4	119.1	O2—C20—H20A	109.5
C6—C5—C4	117.7 (4)	O2—C20—H20B	109.5
C6—C5—C8	121.8 (4)	H20A—C20—H20B	109.5
C4—C5—C8	120.5 (4)	O2—C20—H20C	109.5
O2—C6—C7	123.4 (4)	H20A—C20—H20C	109.5
O2—C6—C5	116.0 (4)	H20B—C20—H20C	109.5
C7—C6—C5	120.6 (4)	O3—C21—H21A	109.5
C6—C7—C2	121.5 (4)	O3—C21—H21B	109.5
C6—C7—H7	119.2	H21A—C21—H21B	109.5
C2—C7—H7	119.2	O3—C21—H21C	109.5
C5—C8—C9	111.5 (3)	H21A—C21—H21C	109.5
C5—C8—H8A	109.3	H21B—C21—H21C	109.5
C9—C8—H8A	109.3	O4—C22—H22A	109.5
C5—C8—H8B	109.3	O4—C22—H22B	109.5
C9—C8—H8B	109.3	H22A—C22—H22B	109.5
H8A—C8—H8B	108.0	O4—C22—H22C	109.5
C28^i^—C28—C27	120.8 (13)	H22A—C22—H22C	109.5
C28^i^—C28—H28A	107.1	H22B—C22—H22C	109.5
C27—C28—H28A	107.1	O5—C23—C24	109.3 (5)
C28^i^—C28—H28B	107.1	O5—C23—H23A	109.8
C27—C28—H28B	107.1	C24—C23—H23A	109.8
H28A—C28—H28B	106.8	O5—C23—H23B	109.8
C28—C27—Br1	125.4 (8)	C24—C23—H23B	109.8
C28—C27—H27A	106.0	H23A—C23—H23B	108.3
Br1—C27—H27A	106.0	C25A—C24—C23	112.8 (7)
C28—C27—H27B	106.0	C23—C24—C25B	116.7 (7)
Br1—C27—H27B	106.0	C25A—C24—H24A	109.0
H27A—C27—H27B	106.3	C23—C24—H24A	109.0
C10—C9—C14	118.2 (4)	C25A—C24—H24B	109.0
C10—C9—C8	120.8 (4)	C23—C24—H24B	109.0
C14—C9—C8	120.9 (4)	H24A—C24—H24B	107.8
O3—C10—C9	115.6 (4)	C23—C24—H24C	108.1
O3—C10—C11	124.0 (4)	C25B—C24—H24C	108.1
C9—C10—C11	120.4 (4)	C23—C24—H24D	108.1
C12—C11—C10	121.9 (4)	C25B—C24—H24D	108.1
C12—C11—H11	119.1	H24C—C24—H24D	107.3
C10—C11—H11	119.1	C26—C25A—C24	139.8 (17)
C11—C12—C13	117.4 (4)	C26—C25A—H25A	110.1
C11—C12—C15	121.6 (4)	C24—C25A—H25A	110.1
C13—C12—C15	121.1 (4)	C26—C25B—C24	129.8 (17)
C14—C13—O4	123.4 (4)	C26—C25B—H25B	115.1
C14—C13—C12	121.2 (4)	C24—C25B—H25B	115.1
O4—C13—C12	115.4 (4)	C25A—C26—H26A	120.0
C13—C14—C9	121.0 (4)	C25A—C26—H26B	120.0
C13—C14—H14	119.5	H26A—C26—H26B	120.0
C9—C14—H14	119.5	C25B—C26—H26C	120.0
C16—C15—C12	112.5 (3)	C25B—C26—H26D	120.0
C16—C15—H15A	109.1	H26C—C26—H26D	120.0
			
C2^i^—C1—C2—C7	−90.9 (4)	C8—C9—C10—C11	−177.2 (3)
C2^i^—C1—C2—C3	87.0 (4)	O3—C10—C11—C12	179.8 (3)
C19—O1—C3—C4	2.2 (6)	C9—C10—C11—C12	−0.2 (6)
C19—O1—C3—C2	−177.7 (4)	C10—C11—C12—C13	−0.1 (5)
C7—C2—C3—C4	0.0 (6)	C10—C11—C12—C15	178.5 (3)
C1—C2—C3—C4	−177.9 (4)	C22—O4—C13—C14	−4.4 (6)
C7—C2—C3—O1	179.8 (4)	C22—O4—C13—C12	176.2 (4)
C1—C2—C3—O1	1.9 (5)	C11—C12—C13—C14	0.3 (6)
O1—C3—C4—C5	−179.5 (4)	C15—C12—C13—C14	−178.4 (3)
C2—C3—C4—C5	0.3 (6)	C11—C12—C13—O4	179.7 (3)
C3—C4—C5—C6	−0.3 (6)	C15—C12—C13—O4	1.0 (5)
C3—C4—C5—C8	177.8 (4)	O4—C13—C14—C9	−179.5 (3)
C20—O2—C6—C7	−15.1 (8)	C12—C13—C14—C9	−0.2 (6)
C20—O2—C6—C5	165.5 (6)	C10—C9—C14—C13	−0.1 (6)
C4—C5—C6—O2	179.6 (4)	C8—C9—C14—C13	177.4 (4)
C8—C5—C6—O2	1.5 (6)	C11—C12—C15—C16	−88.8 (5)
C4—C5—C6—C7	0.1 (6)	C13—C12—C15—C16	89.8 (5)
C8—C5—C6—C7	−178.0 (4)	C12—C15—C16—C18^i^	−88.9 (5)
O2—C6—C7—C2	−179.3 (4)	C12—C15—C16—C17	86.9 (5)
C5—C6—C7—C2	0.1 (6)	C23—O5—C17—C18	5.9 (7)
C3—C2—C7—C6	−0.2 (6)	C23—O5—C17—C16	−174.5 (4)
C1—C2—C7—C6	177.8 (4)	C18^i^—C16—C17—O5	178.5 (4)
C6—C5—C8—C9	90.6 (5)	C15—C16—C17—O5	2.6 (6)
C4—C5—C8—C9	−87.4 (5)	C18^i^—C16—C17—C18	−1.9 (5)
C28^i^—C28—C27—Br1	−173.1 (7)	C15—C16—C17—C18	−177.9 (4)
C5—C8—C9—C10	89.6 (5)	O5—C17—C18—C16^i^	−178.3 (4)
C5—C8—C9—C14	−87.8 (4)	C16—C17—C18—C16^i^	2.2 (6)
C21—O3—C10—C9	−178.1 (4)	C17—O5—C23—C24	177.9 (4)
C21—O3—C10—C11	2.0 (6)	O5—C23—C24—C25A	75.2 (11)
C14—C9—C10—O3	−179.7 (3)	O5—C23—C24—C25B	39.7 (11)
C8—C9—C10—O3	2.8 (5)	C23—C24—C25A—C26	−142.2 (19)
C14—C9—C10—C11	0.3 (5)	C23—C24—C25B—C26	124.1 (18)

Symmetry code: (i) −*x*, *y*, −*z*+1/2.

### Hydrogen-bond geometry (Å, º)

**Table:**

*D*—H···*A*	*D*—H	H···*A*	*D*···*A*	*D*—H···*A*
C27—H27*A*···O2^i^	0.97	3.06	3.82 (1)	136
C27—H27*B*···O4^i^	0.97	3.06	3.99 (1)	160
C28—H28*B*···*Cg*1	0.97	3.10	4.015	158
C28—H28*A*···*Cg*2	0.97	3.28	3.859	120
C19—H19*A*···Br1	0.96	3.14	3.968 (5)	145
C23—H23*A*···Br1	0.97	3.15	4.039 (5)	154

Symmetry code: (i) −*x*, *y*, −*z*+1/2.
